# Central Retinal Artery Occlusion Diagnosed via Ocular Point-of-care Ultrasound: Case Report

**DOI:** 10.5811/cpcem.50741

**Published:** 2026-03-13

**Authors:** Rochelle Kofman, Addison B. Smartt, Reginald J. Myles, Patrick Kishi, Douglas Rappaport, Kevin Drechsel

**Affiliations:** * Mayo Clinic Alix School of Medicine, Phoenix, Arizona; † Mayo Clinic Arizona, Department of Emergency Medicine, Phoenix, Arizona

**Keywords:** point-of-care ultrasound, central retinal artery occlusion, retrobulbar spot sign, cardioembolic stroke, case report

## Abstract

**Introduction:**

Central retinal artery occlusion (CRAO) is a neurological and ophthalmologic emergency that presents as sudden, painless, monocular vision loss. Central retinal artery occlusion can be classified as arteritic or non-arteritic. Most cases of non-arteritic CRAO are due to embolism, commonly from atherosclerosis of the ipsilateral carotid artery. More proximal sources of embolism are uncommon but can occur. Prompt recognition of CRAO is critical for vision preservation therapy and initiation of ischemic stroke diagnosis protocols.

**Case Report:**

We present the case of a 66-year-old female who presented to the emergency department eight hours after sudden, painless, monocular vision loss. Her past medical history included type II diabetes, hypertension, and hyperlipidemia. She had previously undergone bilateral lens replacement for cataracts three years prior and had a history of intermittent floaters, which had been worsening over the previous six months. She denied any associated pain, headache, speech difficulty, focal weakness, and ocular trauma. Point-of-care ultrasound (POCUS) of the affected eye revealed the presence of a retrobulbar spot sign, which is associated with CRAO with a non-arteritic embolic etiology.

**Conclusion:**

Point-of-care ultrasound is an efficient diagnostic tool for the assessment of acute, painless, monocular vision loss. The presence of a retrobulbar spot sign indicates central retinal artery occlusion, providing both diagnostic and prognostic information. Although not a definitive diagnostic tool, POCUS can expedite treatment for patients with central retinal artery occlusion, a diagnosis with a time-sensitive treatment window.

## INTRODUCTION

Central retinal artery occlusion (CRAO) is a rare, vision-threatening neuro-ophthalmologic emergency that presents with sudden, painless, monocular vision loss. Without immediate identification and management, CRAO risks permanent vision loss, with studies showing that > 80% of cases resulted in visual acuity of 20/200 or worse. Better visual outcomes were more common in cases of CRAO with cilioretinal artery sparing.^1^,^2^

The incidence of CRAO in the United States is approximately two cases per 100,000 person-years and increases with male sex and age to 10.1 cases per 100,000 person-years in individuals > 80 years of age.^3^. Central retinal artery occlusion can be classified as arteritic (5%), such as in giant cell arteritis, or non-arteritic (95%).^1^ Most cases of non-arteritic CRAO are due to embolic occlusion of the central retinal artery, commonly from atherosclerosis of the ipsilateral carotid artery, with some studies reporting the presence of carotid artery plaques in up to 71% of cases and significant carotid stenosis in 31–40% of cases.^4^,^5^ Cardiac sources of emboli, such as atrial fibrillation or a cardiac mass, while prevalent, are less common.^6^ There are limited published reports describing cardiac masses, such as fibroelastomas or myxomas, presenting with CRAO as the initial symptom.^7^ This variant highlights the importance of maintaining a broad embolic differential, particularly in patients without known atrial fibrillation or significant carotid disease.

The American Heart Association characterizes CRAO as an acute ischemic stroke event that carries risks of recurrent vascular events.^1^,^8^ Early diagnosis and intervention are critical to avoid irreversible retinal damage and vision loss in addition to identifying and managing underlying vascular risk factors. On examination, fundoscopic findings may reveal a pale retina and a cherry-red spot at the macula. However, these can be subtle or absent in the early presentation. As CRAO is considered a stroke equivalent, standard workup includes immediate neuroimaging, typically with non-contrast head computed tomography (CT) to rule out hemorrhage, followed by CT angiography (CTA) or magnetic resonance imaging (MRI) of the head and neck to assess for vascular occlusion or ischemia. Cardiac telemetry and echocardiography are often used to evaluate for cardioembolic sources such as atrial fibrillation or structural cardiac abnormalities or masses.^1^,^5^

Point-of-care ultrasound (POCUS) plays a valuable role in the rapid evaluation of acute vision loss and has recently emerged as an important adjunct in CRAO diagnosis and management. In particular, the retrobulbar spot sign, a hyperechoic focus within the optic nerve sheath seen on ocular ultrasound, has been associated with CRAO, and its presence has been shown to confirm a thromboembolic cause and can exclude an arteritic etiology. Additionally, this finding also rules out other causes of sudden, painless, monocular vision loss, such as retinal detachment, optic neuropathy, or vitreous hemorrhage and can, therefore, guide further workup and management.^9^–^12^ The sensitivity and specificity of the for detection of an embolic CRAO are 83% and 100%, respectively.^12^

Here we present a case of CRAO in the emergency department (ED) setting, with a retrobulbar spot sign seen on ocular ultrasound. The embolic source was identified by transesophageal echocardiogram as a mass on the mitral valve.

## CASE REPORT

A 66-year-old female presented to the ED eight hours after sudden loss of central vision in the right eye. Her past medical history included diabetes mellitus type II, hypertension, hyperlipidemia, hypothyroidism, and Charcot arthropathy. She had undergone bilateral lens replacement for cataracts three years prior and had a history of intermittent floaters, which had been worsening over the prior six months.

*CPC-EM Capsule*

What do we already know about this clinical entity?

*Central retinal artery occlusion (CRAO) is a neurological and ophthalmologic emergency that presents with painless monocular loss of vision*.

What makes this presentation of disease reportable?

*We present a case of CRAO diagnosed with bedside ocular ultrasound demonstrating retrobulbar spot sign; workup ultimately revealed a cardiac myxoma as the embolic source*.

What is the major learning point?

*CRAO can be diagnosed with ocular ultrasound demonstrating retrobulbar spot sign; further workup can include a bedside echocardiogram to search for an embolic source*.

How might this improve emergency medicine practice?

*Point-of-care ultrasound can aid in a timely diagnosis of CRAO*.

Prior to the sudden and total loss of central vision while sitting at her desk at 9 am that morning, she endorsed seeing “bright lights” in her vision in the preceding few days. She described them as white or gray, occurring transiently. She denied any associated pain, headache, speech difficulty, unilateral weakness, or ocular trauma. On arrival to the ED at 5 pm, she was hypertensive with a blood pressure of 174/74 millimeters of mercury, but her vital signs were otherwise all within normal limits. A neurological examination yielded no focal deficits; she was alert and oriented, pupils were round and reactive, extraocular muscles were intact, and cranial nerves showed no gross deficits. Bilateral conjunctiva and eyelids showed no erythema or injection, and fundoscopic examination yielded poor visualization of the vessels and optic disc.

**Image 1:**
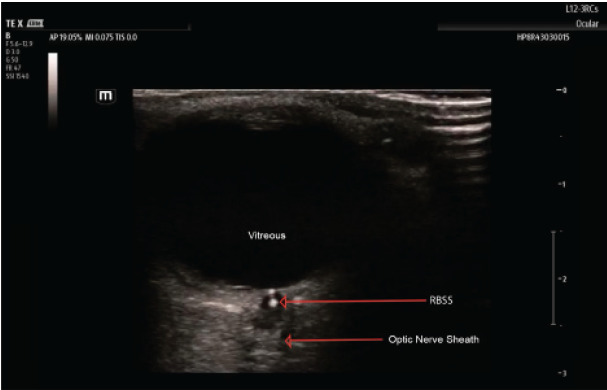
Findings on ocular point-of-care ultrasound using a linear probe demonstrate a retrobulbar spot sign (RBSS) in the transverse view, consistent with central retinal arterial occlusion.

Point-of-care ultrasound (POCUS) of the eye showed no evidence of retinal detachment, vitreous hemorrhage, or posterior vitreous detachment; however, a retrobulbar spot sign was detected (Image 1). This finding in context of the clinical picture put CRAO) at the top of the differential. Based on these findings, a CTA of the head and neck was ordered, and ophthalmology was consulted.

The CTA revealed no acute hemorrhage, ischemia, mass effect, or other gross abnormality. A partially calcified extra-axial mass, likely meningioma, on the left frontal convexity was discovered but was deemed unlikely to be the cause of her vision loss. An exam performed by ophthalmology confirmed central vision loss in the right eye with accompanying macular retinal whitening, with a perfused retinal periphery. This finding combined with the clinical picture pointed toward a CRAO, and the patient’s care was subsequently managed from the perspective of likely ischemic stroke.

**Image 2:**
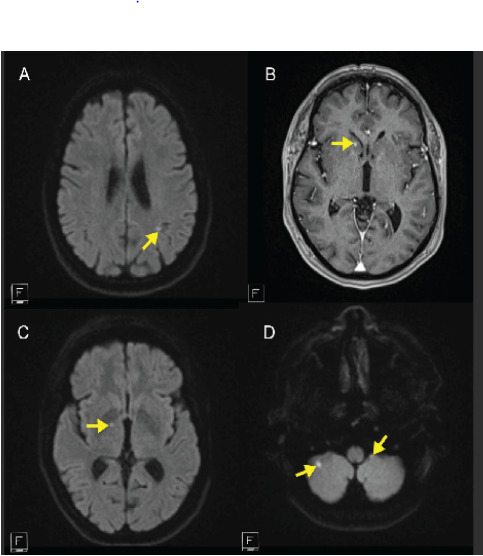
Axial images of brain magnetic resonance imaging obtained with T2-weighted flair (A), T1-weighted post contrast (B), and diffusion-weighted (C, D) sequences showing punctate hyperintensities in the periventricular and deep white matter and cerebellum (yellow arrows) consistent with chronic, small-vessel ischemic changes.

The stroke neurology team recommended brain MRI with and without contrast for further evaluation of both the possibility of ischemic stroke and to further characterize the mass found on CTA. As part of this workup, cardiac telemetry and echocardiography were ordered to assess for a cardioembolic source of emboli. The MRI yielded evidence of multiple, tiny punctate multifocal embolic-appearing acute and subacute infarctions (Image 2). They were present in both the anterior and posterior circulation, indicating a probable proximal source of embolism. This patient was eligible for neither intravenous thrombolysis nor endovascular treatment for the CRAO given the long delay between symptom onset and presentation to the ED.

**Image 3:**
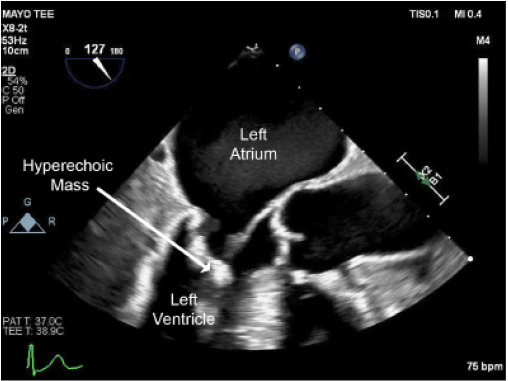
Transesophageal echocardiogram (TEE) midesophageal four-chamber view demonstrating a hyperechoic mass near the mitral annulus (arrow).

**Image 4:**
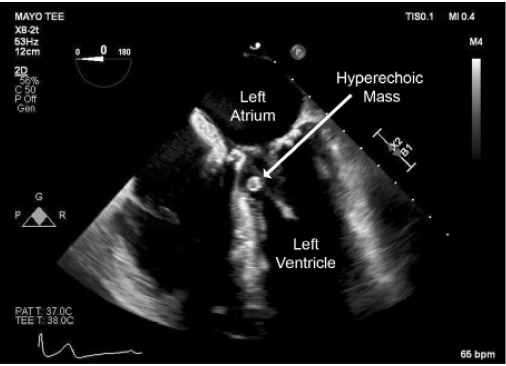
Transesophageal echocardiogram midesophageal long-axis view demonstrating a hyperechoic mass near the mitral annulus (arrow).

On cardiac workup, the electrocardiogram demonstrated normal sinus rhythm; however, a transesophageal echocardiogram performed by cardiology revealed a highly mobile pedunculated mass attached to the mitral valve protruding into the left ventricular outflow tract during systole (Images 3, 4). This finding revealed the source of emboli presenting both clinically and radiographically.

Subsequently, cardiothoracic surgery was consulted and recommended mitral valve replacement and resection of the mass due to its high embolic risk. Endocarditis was ruled out due to lack of fever, leukocytosis, negative blood cultures, and no other signs of infection. She elected to undergo mitral valve mass resection, mitral valve replacement with a tissue valve, and left atrial appendage excision. The operation occurred on day 7 of her admission, delayed for appropriate cessation of anticoagulation. After excision, the mitral valve was determined to have extensive myxoid degeneration and dystrophic calcification by a surgical pathologist.

## DISCUSSION

Patients with CRAO typically present with sudden, painless vision loss. The differential for this presentation, however, can be wide, including retinal detachment, vitreous bleeding, temporal arteritis, and optic neuropathy. Central retinal artery occlusion visualized with ultrasound may show a hyperechoic retrobulbar spot sign, which represents embolic activity in the retinal artery. The retrobulbar spot sign can also show papilledema.

When assessing for a retrobulbar spot sign there is risk of optic nerve drusen presenting as a false positive for CRAO. This deposit of hyalin calcific material in the optic nerve head may cause similar symptoms but can be excluded given clinical context and accurate capture with ultrasound. Optic nerve drusen is an insidious visual impairment that develops chronically. Because of this, optic nerve drusen is often found incidentally. Both size and location can differentiate optic nerve drusen from CRAO with retrobulbar spot sign, given that ultrasound visualization of optic nerve drusen is larger and present at the terminal optic nerve,^13^ while the retrobulbar spot sign is smaller and presents more centrally and further from the optic nerve ending.^10^

Point-of-care ultrasound can be an efficient diagnostic tool to narrow the differential of acute, painless, monocular vision loss. Non-ionizing imaging is safe and presents no risk of radiation exposure. The use of Doppler can indicate abnormalities in blood flow, aiding in differentiating CRAO from other diagnoses. As documented in McGuire et al, POCUS with the use of Doppler can also be used to monitor progression with serial Doppler measurements, tracking the patient’s progression over time.^14^ These functions make POCUS evaluation of CRAO especially useful in emergent cases and situations where ophthalmic specialists are not immediately available. Additionally, there is evidence that the presence of a retrobulbar spot sign predicts poor response to thrombolytic treatment for CRAO.^15^

However, POCUS has clear limitations. There can be discomfort associated with pressure applied to the eye. However, there is no evidence of ultrasound use causing damage to the retina or optic nerve when performed with low-intensity, non-contact techniques. Accuracy of Doppler evaluation is heavily dependent on user expertise, as there is a small margin of error in the angle of visualization. If the angle is off slightly, the signal and strength of the Doppler flow will decrease. Additionally, the absence of a retrobulbar spot sign does not rule out a diagnosis of CRAO. For these reasons, ultrasound is not the definitive diagnostic tool for vision loss or CRAO, although it can provide timely, accurate, and inexpensive diagnostic information.

Given the negligible risk profile and significant diagnostic utility of ultrasound, especially in the acute setting where rapid differentiation from other causes of acute vision loss is essential, the benefits of ultrasound in diagnosing CRAO far outweigh any potential adverse effects. The use of POCUS in this patient’s workup revealed the retrobulbar spot sign of the right eye, indicating CRAO. Point-of-care ultrasound narrowed the differential and greatly expedited further exploration of the embolic source, leading to subsequent discovery and excision of this patient’s cardiac myxoma. Although in this case the patient was not eligible for thrombolytic treatment, expedited workup and diagnosis of CRAO with POCUS may preserve the possibility of thrombolysis for a patient presenting earlier with this diagnosis. This case affirms the utility of POCUS in patients presenting with acute, monocular vision loss, both for narrowing the diagnostic differential and expediting subsequent treatment.

## CONCLUSION

Point-of-care ultrasound is an efficient and effective diagnostic tool for the assessment of acute, painless, monocular vision loss. The presence of a retrobulbar spot sign indicates central retinal artery occlusion, providing both diagnostic and prognostic information. Although not a definitive diagnostic tool, POCUS can expedite treatment for patients with CRAO, a diagnosis with a time-sensitive treatment window.

## References

[1] Mac Grory B, Schrag M, Biousse V (2021). Management of central retinal artery occlusion: a scientific statement from the American Heart Association [published correction appears in *Stroke*. 2021 ;52(6):e309]. Stroke.

[2] Kim YH, Park KH, Woo SJ (2020). Clinical manifestations and visual prognosis of cilioretinal artery sparing central retinal artery occlusion. Korean J Ophthalmol.

[3] Leavitt JA, Larson TA, Hodge DO, Gullerud RE (2011). The incidence of central retinal artery occlusion in Olmsted County, Minnesota. Am J Ophthalmol.

[4] Hayreh SS, Podhajsky PA, Zimmerman MB (2009). Retinal artery occlusion: associated systemic and ophthalmic abnormalities. Ophthalmology.

[5] Tiwari V, Bagga SSJ, Prasad R, Mathurkar S (2024). A review of current literature on central retinal artery occlusion: its pathogenesis, clinical management, and treatment. Cureus.

[6] Mac Grory B, Landman SR, Ziegler PD (2021). Detection of atrial fibrillation after central retinal artery occlusion. Stroke.

[7] Ma JH, Gill MK (2018). Calcified amorphous tumor: a rare cause of central retinal artery occlusion. Am J Ophthalmol Case Rep.

[8] Chodnicki KD, Pulido JS, Hodge DO, Klaas JP, Chen JJ (2019). Stroke risk before and after central retinal artery occlusion in a US cohort. Mayo Clin Proc.

[9] Smith AT, Wilbert CD, Ferre RM (2020). Using the retrobulbar spot sign to assist in diagnosis and management of central retinal artery occlusions. J Ultrasound Med.

[10] Lottspeich C, Mackert MJ, Köhler A, Bauer A, Hoffmann U, Czihal M (2021). Retrobulbar spot sign in metachronous bilateral central retinal artery occlusion of cardioembolic origin. J Neuroophthalmol.

[11] Czihal M, Lottspeich C, Köhler A (2021). Transocular sonography in acute arterial occlusions of the eye in elderly patients: diagnostic value of the spot sign. PLoS One.

[12] Ertl M, Altmann M, Torka E (2012). The retrobulbar “spot sign” as a discriminator between vasculitic and thrombo-embolic affections of the retinal blood supply. Ultraschall Med.

[13] Almog Y, Nemet A, Nemet AY (2016). Optic disc drusen demonstrate a hyperechogenic artifact in B mode ultrasound. J Clin Neurosci.

[14] McGuire D, Calleja R, Pai E, Bahl A (2024). Emergency department Doppler assessment of a central retinal artery occlusion: case report. Clin Pract Cases Emerg Med.

[15] Nedelmann M, Graef M, Weinand F (2015). Retrobulbar spot sign predicts thrombolytic treatment effects and etiology in central retinal artery occlusion. Stroke.

